# Comparative Metabolomics Analysis of Different Perilla Varieties Provides Insights into Variation in Seed Metabolite Profiles and Antioxidant Activities

**DOI:** 10.3390/foods12234370

**Published:** 2023-12-04

**Authors:** Senouwa Segla Koffi Dossou, Qianchun Deng, Feng Li, Nanjun Jiang, Rong Zhou, Lei Wang, Donghua Li, Meilian Tan, Jun You, Linhai Wang

**Affiliations:** 1 Key Laboratory of Biology and Genetic Improvement of Oil Crops, Ministry of Agriculture and Rural Affairs, Oil Crops Research Institute, Chinese Academy of Agricultural Sciences, Wuhan 430062, Chinajunyou@caas.cn (J.Y.); 2 Amway (China) Botanical R&D Center, Wuxi 214115, China

**Keywords:** perilla, metabolite profiling, antioxidant activity, bioactive compounds, metabolic markers, seed coat color, LC-MS

## Abstract

Perilla seeds are essential functional foods and key ingredients in traditional medicine. Herein, we investigated the variation in phytochemical profiles and antioxidant activities of twelve different perilla seeds. The seeds showed significant variations in total phenolic and flavonoid contents ranging from 16.92 to 37.23 mg GAE/g (GAE, gallic acid equivalent) and 11.6 to 19.52 mg CAE/g (CAE, catechin equivalent), respectively. LC-QqQ-MS (liquid chromatography triple quadrupole tandem mass spectrometry)-based widely targeted metabolic profiling identified a total of 975 metabolites, including 68–269 differentially accumulated metabolites (DAMs). Multivariate analyses categorized the seeds into four groups based on the seed coat and leaf colors. Most key bioactive DAMs, including flavonoids (quercetin-3’-*O*-glucoside, prunin, naringenin, naringenin chalcone, butin, genistin, kaempferol-3-*O*-rutinoside, etc.), amino acids (valine, lysine, histidine, glutamine, threonine, etc.), and vitamins (B1, B3, B6, U, etc.) exhibited the highest relative content in PL3 (brown seed, purple leaf), PL1 (white seed, green-purple leaf), and PL4 (white seed, green leaf) groups, respectively. Meanwhile, key differentially accumulated phenolic acids showed a higher relative content in PL1 and PL4 than in other groups. Both seeds exhibited high antioxidant activities, although those of PL2 (brown seed, green leaf) group seeds were the lowest. Our results may facilitate the comprehensive use of perilla seeds in food and pharmaceutical industries.

## 1. Introduction

A member of the Labiatae family, *Perilla frutescens* is an important nutraceutical and pharmaceutical crop grown principally in East Asian countries [1,2]. It is an essential therapeutical and economic herb that is commonly appropriated in Korea, China, Thailand, Japan, Vietnam, India, and Taiwan. Historically, different parts of the perilla plant, including leaf, seed, and stem, have been equally used as drugs since ancient times (in the Song dynasty from 960 to 1279 A.D), and the herb has been recorded in the Chinese medical classics since around 500 AD [3]. Studies have demonstrated that perilla possesses diverse biological attributes, such as anticancer, antioxidant, antimicrobial, anti-inflammatory, anti-depressive [2,3,4,5,6,7,8,9], anti-osteoporosis [10], enhancement of cognitive function in elderlies [11,12], and anti-SARS-CoV2 [13]. Accordingly, perilla has taken an important part of people’s daily life and has captivated scientists’ interest in analyzing its phytochemical composition, metabolite diversity, and bioactivities, prompting its growing popularity worldwide [14].

Three types of perilla, including green, red (purple), and red/green, are mainly found based on the leaf phenotypes [3], inferring a diversity and variation of metabolites for various applications. However, although the leaves, stem, and seeds are widely used in traditional medicine, most studies have been concentrated on the leaf’s chemical composition, while few studies have focused on the seeds’ metabolite diversity and variability. Therefore, a thorough examination of the diversity and variability of perilla seed metabolites will provide fundamental resources for their comprehensive use. Widely targeted metabolomics profiling is one of the most advanced tools for deciphering the chemical components of the metabolome of plant organs. It allows for an understanding of the diversity in plant phenotypes via an accurate identification and evaluation of the relative contents of all metabolites and the exploration of metabolite profile changes in different plant parts [15,16,17,18,19,20]. It has been used to differentiate between the metabolite profiles of sesame seeds of different colors and unveil the differentially accumulated metabolites (DAMs) and pathways [16]. With the availability of perilla genome sequence information [21], identifying biochemical markers for discriminating between perilla seeds will facilitate the dissection of the genetic basis underlying variations in major bioactive compounds. 

Previous research on perilla seeds was mainly related to oil content and composition [22,23]. Phytochemical characterization studies have revealed that the perilla seed is of great nutritional quality and contains diverse natural antioxidant compounds, such as flavonoids, phenolic acids, tocopherols, phytosterols, polycosanols, squalene, etc. [1,22]. Consequently, perilla seed oil is extensively used in cosmetics and to produce healthcare drugs in China [22]. Unfortunately, a few metabolites in perilla seed have been identified and structurally characterized [1,14,24]. Among them, four phenolic compounds, including rosmarinic acid, luteolin, rosmarinic acid-3-*O*-glucoside, and apigenin, have been identified as the perilla seed biomarkers [24]. Regarding different perilla seed types, the study conducted by Kongkeaw et al. revealed that brown seeds had significantly higher protein, ash, fat, crude fiber, minerals (Ca, Mg, and P), polyunsaturated fatty acids (γ-linolenic acid and α-linolenic acid), β-carotene, α-tocopherol, total phenolic, total flavonoid, and total flavonol contents and antioxidant capability than white seeds [25]. Therefore, a comparative analysis of the metabolite profiles of different types of perilla seeds will generate important data that may be useful for obtaining nutritional and medicinal value from perilla.

In this study, we applied UHPLC-ESI-QqQLIT-MS/MS (ultra-high-performance liquid chromatography coupled with electrospray ionization tandem triple quadrupole mass spectrometry) to reveal the metabolite profiles of different perilla seeds. Through multivariate and functional analyses, we unveiled DAMs and differentially regulated pathways. In addition, we evaluated the total phenolic and flavonoid contents and antioxidant activities of the different perilla seeds. Our results provide valuable data and theoretical guidance for the scientific-basis use of perilla seeds.

## 2. Materials and Methods

### 2.1. Plant Materials and Chemical Reagents

**Figure 1:**
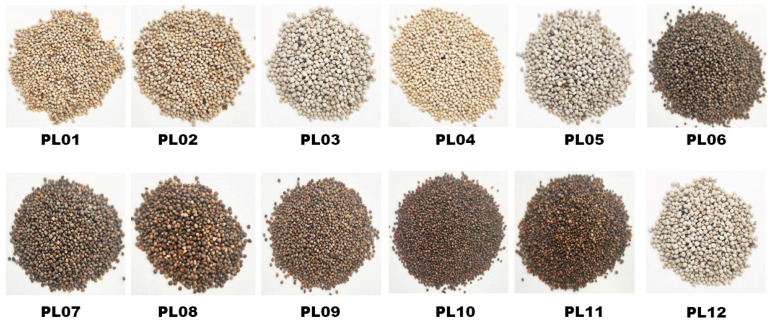
Images of the different perilla seeds analyzed in the present study. The sample labels are defined in Table 1.

Twelve perilla varieties (Table 1, Figure 1) offered by the Oil Crops Research Institute of the Chinese Academy of Agricultural Sciences (OCRI-CAAS), Wuhan, China, were analyzed in this study. The varieties are mainly different in seed coat and leaf color (Table 1). The seeds were grown from June to September 2022 at an experimental field station of OCRI-CAAS located in Wuhan, China (N 30.57°, E 114.30°, 27 m altitude), under identical environmental conditions. Seed samples were collected 30 days after flowering, with three replications for each genotype. Each sample was constituted by a mixture of seeds from ten individual plants. All samples were directly frozen in liquid nitrogen and conserved at −80 °C until the metabolomic profiling analysis five weeks later. Another group of samples was prepared similarly, dried in the sun to water content of 9–10%, and kept at the OCRI seeds room until the evaluation of the total phenolic content, flavonoid content, and antioxidant activities three months later.

The LC-MS gradient grade solvents, including methanol, acetic acid, and acetonitrile, were purchased from Merck Company (Darmstadt, Germany). All other chemicals and standards of metabolites were purchased from Sigma-Aldrich (St. Louis, MO, USA) or BioBioPha (Kunming, China). Each standard was dissolved in dimethyl sulfoxide or methanol (standard stock solutions) and stored at −20 °C. Before the MS analysis, all standard stock solutions were diluted with 70% methanol to constitute a gradient of different concentrations.

### 2.2. Evaluation of Total Phenolic (TPC) and Flavonoid (TFC) Contents

Seed samples (0.5 g) were extracted with 5 mL 80% ethanol (constant shaking in darkness) for 4 h, followed by centrifugation at 5000 g for 15 min [26,27]. The supernatants were collected, filtered, completed to 5 mL with 80% ethanol, and stored at −20 °C for further analyses within a period of one week. The TPC and TFC were evaluated per the method outlined by Choi et al. [27]. For the TPC, 100 μL of each seed extract was mixed with 400 μL dH_2_O and 100 μL Folin-Ciocalten reagent. Then, 6 min later, 1 mL of 7% (*m*/*v*) Na_2_CO_3_ was added, followed by 800 μL of dH_2_O. Next, the mixture was kept for 90 min at room temperature. The absorbance of all reaction solution was recorded against the blank (80% ethanol) at 760 nm with a spectrophotometer (UV5200, Shanghai Metash Instruments Co., Ltd., Shanghai, China). The TPC was expressed as mg GAE (gallic acid equivalents) per gram of seeds. The regression equation was y = 1.971x − 0.0068 (R^2^ = 0.99).

For TFC, 1 mL of each seed extract was mixed with 150 μL of 5% (*m*/*v*) NaNO_2_. After 6 min, 300 μL of 10% (*m*/*v*) AlCl_3_·6H_2_O was added, followed by another incubation for 6 min. Next, 1 mL of 1 M NaOH and 1.05 mL dH_2_O were added. Finally, we mixed the solutions well and recorded the absorbance at 510 nm immediately. The TFC was computed from a standard curve for catechin and expressed as mg CAE/g (catechin equivalent per gram) seeds. The regression equation was y = 3.253x + 0.1447, (R² = 0.9702).

### 2.3. Sample Preparation and Extraction

Freeze-dried seed samples (moisture content of 9–10%) were crushed for 1.5 min at 30 Hz with a mixer mill (MM 400, Retsch, Haan, Germany). Then, lyophilized powder (100 mg) of each sample was extracted overnight at 4 °C with 1.2 mL of 70% methanol, followed by 15 min centrifugation at 12,000 g. Subsequently, the supernatants were collected separately and filtrated through a 0.22 μm micropore membrane (SCAA-104, ANPEL, Shanghai, China). All extracts were kept at −20 °C up to the UHPLC-ESI-QqQLIT-MS/MS analysis [16,17,28]. All 36 sample extracts were mixed equally to constitute quality control (QC) samples. 

### 2.4. Data Acquisition and Multivariate Analyses

The metabolomics and multivariate analyses were performed as we previously reported at Novogene Co., Ltd., (Beijing, China) [16,17]. Briefly, the data acquisition system was composed mainly of a UHPLC system (Shim-pack UFLC SHIMADZU CBM30A) and a tandem mass spectrometry (MS/MS) system (Applied Biosystems 6500 QTRAP). Metabolites were qualitatively identified based on spectrum information, retention times, and mass spectra. The relative content of each metabolite was computed via the multiple reaction monitoring (MRM) modes that consisted of triple quadrupole (QqQ) MS analyses. The multivariate analyses were performed after data quality assessment and standardization via Zscore. The PCA (principal component analysis), HCA (hierarchical clustering analysis), and OPLS-DA (orthogonal partial least squares discriminant analysis) were carried out in R (version 3.5.0) using the statistics packages prcomp, pheatmap, and MetaboAnalystR (www.r-project.org), respectively. Significant DAMs (differentially accumulated metabolites) were screened out at thresholds of fold-change (FC ≥ 2 or ≤0.5), VIP ≥ 1, and *p*-value < 0.05. The VIP (variable important in projection) values were extracted from the OPLS-DA analysis. Finally, the functional annotation of DAMs was conducted through mapping to the KEGG (Kyoto Encyclopedia of Genes and Genomes) database and subsequent significant enrichment analyses using MSEA (metabolite sets enrichment analysis). The hypergeometric test’s *p*-values were used to filter significantly enriched pathways.

### 2.5. Antioxidant Activity of the Different Seeds

To determine the antioxidant activities of the seeds, we carried out DPPH and FRAP assays as reported in our recent publications [16,29].

### 2.6. Statistical Analysis 

GraphPad Prism v9.0.0121 (GraphPad 159 Software Inc., La Jolla, CA, USA) was used for graph construction, and TBtools software (version 1.9) was used for heatmapping [30]. ANOVA (analysis of variance) test was used for comparison, and statistically differences were determined at *p* ˂ 0.05. 

## 3. Results

### 3.1. Total Phenolic (TPC) and Total Flavonoid (TFC) Contents of the Twelve Perilla Seeds

**Figure 2:**
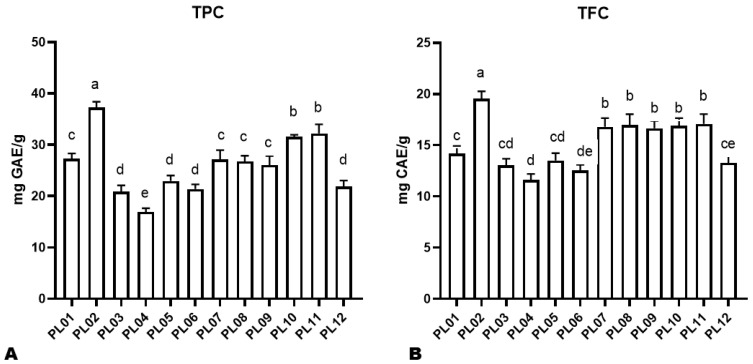
Total phenolic content (**A**) and total flavonoid content (**B**) of the twelve different perilla seeds. The sample labels are defined in Table 1. Different letters above bars indicate statistically significant differences at *p* ˂ 0.05.

To explore the potential of the twelve perilla seeds, we investigated their TPC and TFC. As shown in Figure 2, we observed a significant variation in the TPC and TFC among the twelve different perilla seeds. For instance, the TPC and TFC varied from 16.92 (PL04) to 37.23 mg GAE/g (PL02) and 11.6 (PL04) to 19.52 mg CAE/g (PL02), respectively (Figure 2A,B). Seeds of varieties PL07, PL08, and PL09 exhibited a similar TPC and TFC (Figure 2A,B).

### 3.2. Metabolite Profiles of Perilla Seeds

To gain insight into the diversity and variation of metabolites in perilla seeds, the twelve varieties (Table 1, Figure 1) were subjected to UHPLC-ESI-QqQLIT-MS/MS-based widely targeted metabolomics analysis [17,28]. In total, 975 metabolites, including 413 and 562 in negative and positive ions, respectively, were detected and chemically characterized (Table S1).

**Figure 3:**
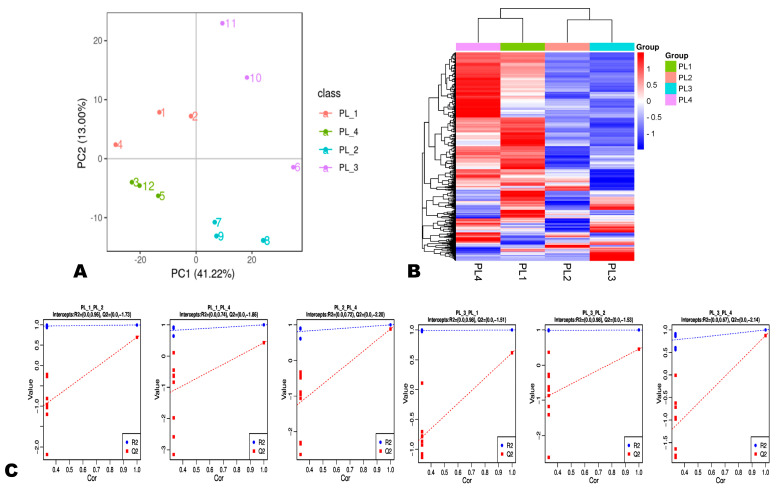
Overview of the diversity and variation of metabolites in perilla seeds. (**A**) Score plot of principal component analysis (PCA) results showing metabolite profile differences between and within groups. (**B**) Hierarchical clustering analysis results; the profiles of all types of metabolites were normalized to complete the hierarchical clustering. Each group is represented by one column, and each metabolite is visualized in one row. Red and blue indicate high and low abundance, respectively. (**C**) OPLS-DA results of pairwise comparison between groups of perilla seeds. The sample labels are defined in Table 1.

Principal component analysis (PCA) and hierarchical cluster analysis (HCA) are important analytical methods that enable the examination of metabolites’ variability among different samples. The PCA showed that the twelve seed samples could be categorized into four groups according to their metabolite profiles (Figure 3A). Notably, the different constituted groups, including PL1 (white seed, green-purple leaf, including PL01, PL02, and PL04), PL2 (brown seed, green leaf, including PL07, PL08, and PL09), PL3 (brown seed, purple leaf, including PL06, PL10, and PL11), and PL4 (white seed, green leaf, including PL03, PL05, and PL12) could be distinguished by the seed colors and the leaf colors of the varieties (Table 1, Figure 1). Except for PL4, different varieties in PL1, PL2, and PL3 gathered not very closely, indicating that the metabolite profile of perilla seed may vary depending on the variety (Figure 3A). As presented in Figure 3B, the HCA confirmed the variability of metabolites among the different groups. The seeds with white coats exhibited a higher relative content of many identified metabolites. To confirm the observed variation in metabolite profiles, we further carried out an OPLS-DA analysis, and the results were supportive, with a strong goodness of fit and high predictability (Figure 3C). 

### 3.3. Classification and Variation of Major Metabolite Classes in Perilla Seeds

**Figure 4:**
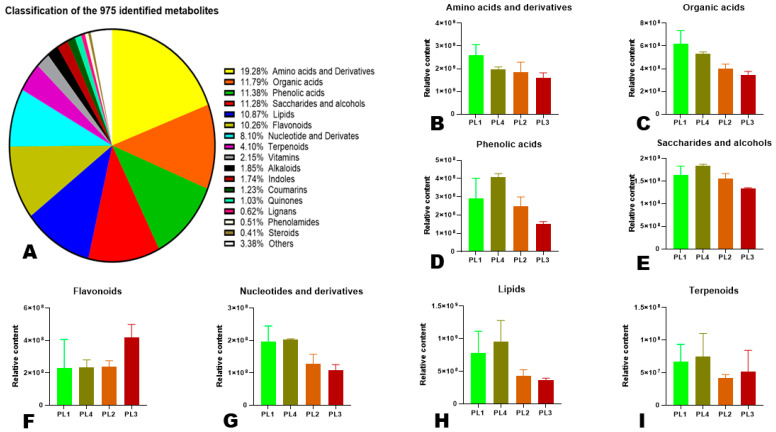
Classification and variation of metabolites. (**A**) Classification of the 975 identified metabolites in perilla seeds. (**B**–**I**) Relative content of major classes of metabolites in the different groups of perilla seeds. The sample labels are defined in Table 1.

The classification of the identified metabolites revealed that the major classes of metabolites in perilla seeds were amino acids and derivatives (19.28%), organic acids (11.79%), phenolic acids (11.38%), saccharides and alcohols (11.28%), lipids (10.87%), flavonoids (10.26%), and nucleotides and derivatives (8.10%) (Figure 4A). To examine the variation of the relative content of major metabolite classes in the different groups of perilla seeds, we computed the sum of all intensities and constructed bar graphs of the predominant metabolite classes (Figure 4B–I). It showed that white perilla seeds (PL1 and PL4) are rich in amino acids and derivatives, organic acids, phenolic acids, saccharides and alcohols, nucleotides and derivatives, lipids, and terpenoids than brown seeds (Figure 4B–E,G–I). In contrast, the brown perilla seeds exhibited a higher relative content of flavonoids than the white seeds (Figure 4F).

### 3.4. Differentially Accumulated Metabolites (DAMs) and Functional Annotation

**Figure 5:**
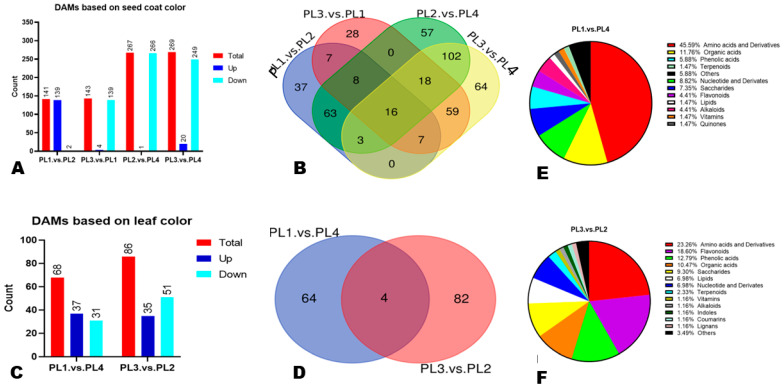
Differentially accumulated metabolites (DAMs) between perilla seeds of different genotypes. (**A**) Number of DAMs in pairwise comparison between perilla seeds of different colors. (**B**) Venn diagrams showing the number of overlapping DAMs between perilla seeds of different colors. (**C**) Number of DAMs in pairwise comparison between perilla seeds from genotypes different in leaf color. (**D**) Venn diagrams showing the number of overlapping DAMs. (**E**) Classification of DAMs in pairwise comparison of PL1. vs. PL4. (**F**) Classification of DAMs in pairwise comparison of PL3. vs. PL2. The sample labels are defined in Table 1.

In order to unravel the impact of seed coat color and leaf color on metabolite variation in perilla seeds, we performed a DAMs analysis. Significant DAMs in a pairwise comparison between groups were uncovered at thresholds of fold-change (FC ≥ 2 or ≤ 0.5), VIP ≥ 1, and *p*-value < 0.05, and the volcano plots are shown in Figures S1 and S2. Based on the difference in seed coat color, we detected a total of 141 (139 up-regulated in PL1) and 143 (139 up-regulated in PL1) significant DAMs in a pairwise comparison between PL1. vs. PL2 and PL3. vs. PL1, respectively (Figure 5A). Meanwhile, in the pairwise comparison of PL4 against PL2 and PL3, respectively, there were 267 (266 up-regulated in PL4) and 269 (249 up-regulated in PL4) significant DAMs (Figure 5A). To identify key metabolites for discriminating between perilla seeds of different colors, we constructed a Venn diagram among the detected DAMs (Figure 5B). The results revealed that sixteen metabolites belonging to diverse classes overlapped (Table S2).

To investigate the influence of leaf color on the variation in perilla seeds’ metabolites, we focused on pairwise comparisons of PL1. vs. PL4 and PL3. vs. PL2. We detected 68 (37 up-regulated in PL1) and 86 (35 up-regulated in PL3) DAMs between PL1. vs. PL4 and PL3. vs. PL2, respectively (Figure 5C). The lists are presented in Tables S3 and S4. Only four DAMs were common to the two pairwise comparisons, indicating that leaf color greatly influences seed metabolite composition (Figure 5D). The five major classes of DAMs between PL1 (white seed, green-purple leaf) and PL4 (white seed, green leaf) were amino acids and derivatives (45.59%), organic acids (11.76%), nucleotides and derivatives (8.82%), saccharides (7.35%), and phenolic acids (5.88%) (Figure 5E). Meanwhile, amino acids and derivatives (23.26%), flavonoids (18.60%), phenolic acids (12.79%), organic acids (10.47%), and saccharides (9.30%) were the five major classes of DAMs between PL2 (brown seed, green leaf) and PL3 (brown seed, purple leaf) (Figure 5F).

To unveil the differentially regulated pathways, we conducted a KEGG analysis of the DAMs (Figures S3 and S4). The DAMs between PL1 and PL2 were mainly assigned to the pentose phosphate pathway, nicotinate and nicotinamide metabolism, fructose and mannose metabolism, carbon metabolism, phenylpropanoid biosynthesis, and phenylalanine metabolism (Figure S3A). Meanwhile, the DAMs between PL3 and PL1 were primarily involved in ABC transporters; 2-oxocarboxilic acid metabolism; glutathione metabolism; arginine and proline metabolism; glyoxylate and dicarboxylate metabolism; alanine, aspartate, and glutamate metabolism; citrate cycle; and C5-branched dibasic acid metabolism (Figure S3B). Similarly, the DAMs in a pairwise comparison of PL4 against PL2 and PL3, respectively, were assigned to different metabolic pathways (Figure S4A,B). Regarding the leaf color, the results indicated that the DAMs between PL1 and PL4 were assigned to ABC transporters, pyrimidine metabolism, histidine metabolism, arginine biosynthesis, purine metabolism, and aminoacyl-tRNA biosynthesis (Figure S3C). In contrast, the DAMs between PL2 and PL3 were mainly involved in the biosynthesis of amino acids, flavonoid biosynthesis, 2-oxocarboxylic acid metabolism, arginine and proline metabolism, and glutathione metabolism (Figure S3D).

### 3.5. Variation Characteristics of Key Bioactive DAMs in Perilla Seeds

**Figure 6:**
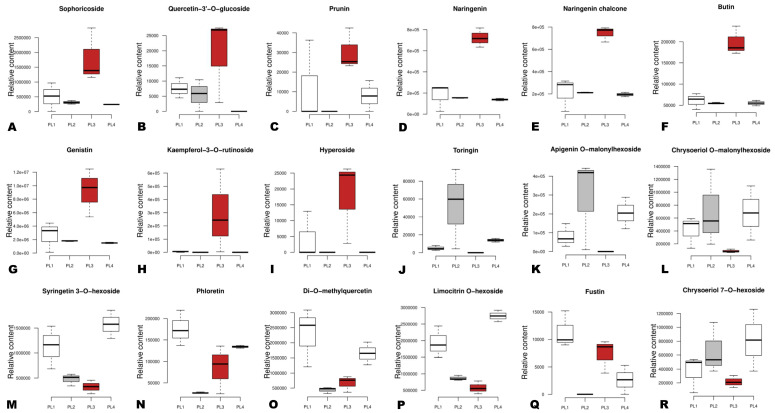
Box plots showing the variation of key differentially accumulated bioactive flavonoids in the different perilla seeds. (**A**–**R**) The specific metabolite is written above of each graph. The sample labels are defined in Table 1.

To explore the variation of bioactive compounds in the different perilla seeds, we examined the relative content of 72 DAMs, including 18 flavonoids, 24 phenolic acids, 14 amino acids, 9 vitamins, 2 alkaloids, 2 quinones, and 3 terpenoids. As shown in Figure 6, nine flavonoids, including sophoricoside, quercetin-3’-O-glucoside, prunin, naringenin, naringenin chalcone, butin, genistin, kaempferol-3-O-rutinoside, and hyperoside, exhibited the highest relative content in PL3. Apigenin O-malonylhexoside and toringin showed higher contents in PL2 than other seeds (Figure 6). PL1 and PL4 (white seeds) exhibited the highest relative content of phloretin, syringetin 3-O-hexoside, di-O-methylquercetin, and limocitrin O-hexoside (Figure 6).

**Figure 7:**
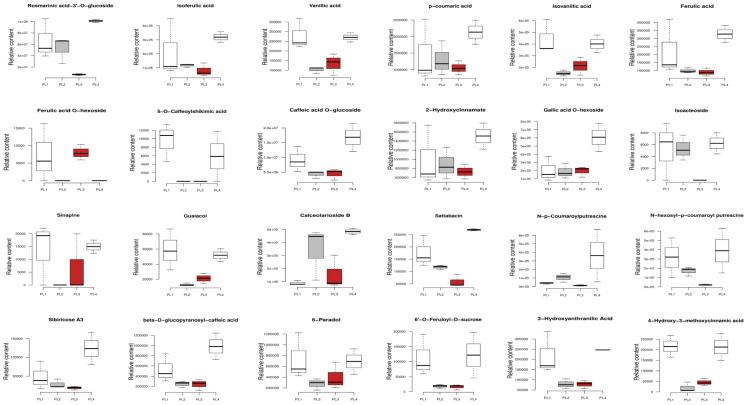
Box plots showing the variation in the relative content of key differentially accumulated bioactive phenolic acids in the different perilla seeds. The specific metabolite is written above of each graph. The sample labels are defined in Table 1.

All the phenolic acids (rosmarinic acid-3’-O-glucoside, isovanillic acid, 2-hydroxycinnamate, 4-hydroxy-3-methoxycinnamic acid, 5-O-caffeoylshikimic acid, caffeic acid O-glucoside, isoacteoside, p-coumaric acid, vanillic acid, ferulic acid, etc.) exhibited the highest relative content in white seeds, especially in PL4, except for ferulic acid O-hexoside (Figure 7). The relative content of trigonelline (alkaloid) and valepotriate (terpenoid) in PL1 was the highest (Figure S5). PL4 exhibited the highest relative content of tabersonine (alkaloid), Kynurenic acid (quinone), and geniposidic acid (terpenoid) (Figure S5). The relative content of madecassic acid (terpenoid) and bisulfurous acid (quinone) was the highest (Figure S5). 

**Figure 8:**
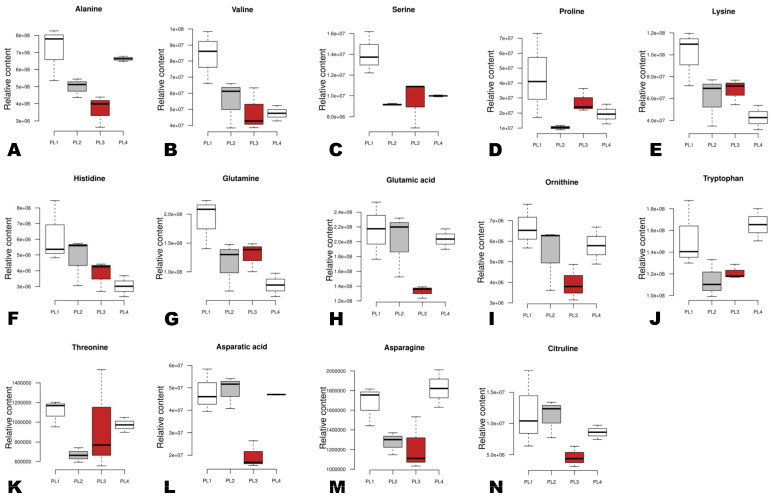
Box plots showing the variation of key differentially accumulated bioactive amino acids in the different perilla seeds. (**A**–**N**) The specific metabolite is written above of each graph. The sample labels are defined in Table 1.

**Figure 9:**
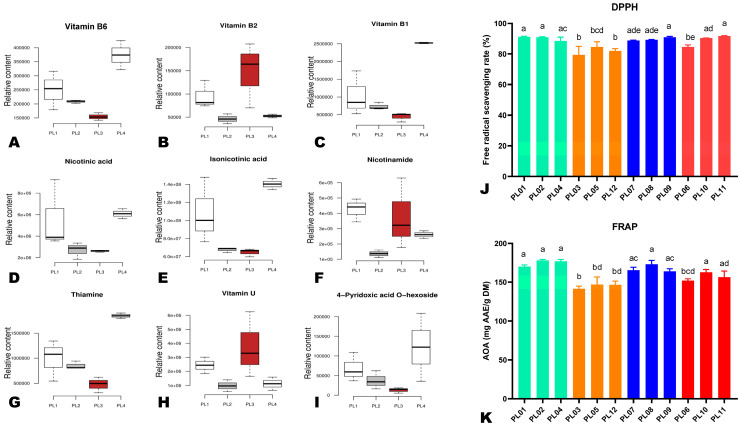
(**A**–**I**) Box plots showing the variation in the relative content of key differentially accumulated bioactive vitamins in the different perilla seeds. The specific metabolite is written above of each graph. (**J**,**K**) Antioxidant activities of the different perilla seeds via DPPH and FRAP assays, respectively. Different colored bars indicate the different groups, including PL1 (PL01, PL02, and PL04), PL2 (PL07, PL08, and PL09), PL3 (PL06, PL10, and PL11), and PL4 (PL03, PL05, and PL12). The sample labels are defined in Table 1. Different letters above the bars indicate statistically significant differences at *p* ˂ 0.05.

Most of the examined amino acids (alanine, valine, serine, proline, lysine, histidine, glutamine, threonine, ornithine, and glutaric acid) showed a higher relative content in PL1 than in other seeds, except for tryptophan, asparagine, aspartic acid, and citrulline (Figure 8). Tryptophan and asparagine exhibited the highest relative content in PL4, followed by PL1 (Figure 8). Regarding vitamins, vitamin B6, vitamin B1, nicotinic acid, isonicotinic acid, thiamine, and 4-pyridoxic acid O-hexoside exhibited the highest relative content in PL4, followed by PL1, PL2, and PL3 (Figure 9A–I). Meanwhile, the relative content of vitamin B2 and vitamin U in PL3 was the highest, followed by PL1 (Figure 9A–I).

### 3.6. Antioxidant Activity of the Different Perilla Seeds

To integrate the variation in metabolite profiles and the bioactivity of the different perilla seeds, we carried out the antioxidant activity assays. The results showed that the different perilla seeds had a higher antioxidant capability (Figure 9J,K). However, compared to other groups, samples in the PL4 group (PL03, PL05, and PL12) exhibited significantly lower antioxidant activities (Figure 9J,K). 

## 4. Discussion

Perilla is a prominent oilseed crop grown mainly in East Asia, where it occupies a place of choice in traditional medicine [4]. As an essential ingredient in food pharmacy, perilla has been classified as “One Root of Medicine and Food” for its high nutritional values and recorded pharmacological attributes [2,11,14,31]. All the aboveground organs of the plant are generally equally used as drugs. However, a few studies have focused on seed phytochemical profiles, and the diversity and variability of metabolites in perilla seeds have been elusive. Thus, the present study applied a widely targeted metabolomics approach to reveal the metabolite profiles of four groups of perilla seeds different in coat and leaf colors. Metabolomics profiling is an advanced analytical method used to identify DAMs between different groups of plant organs and to elucidate the correlations between phenotypes and biological processes [16,32]. 

Seed metabolites represent the primary source of nutrients for livestock and humans or a supply of key raw materials for various industries [33]. Previous studies have revealed that perilla seeds contain diverse classes of metabolites, including lipids, phenolic acids, flavonoids, phytosterols, tocopherols, policosanols, etc. [1,14,22]. However, the maximum number of metabolites identified in perilla seeds included 57 and 105 nonvolatile and volatile chemical components, respectively [14]. Herein, we identified 975 metabolites classified into diverse classes, of which amino acids and derivatives, organic acids, phenolic acids, saccharides and alcohols, lipids, flavonoids, and nucleotides and derivatives were dominant. Multivariate analyses revealed a considerable variation of metabolites among varieties and groups. We detected 141, 143, 267, and 269 DAMs in pairwise comparisons between white (PL1 and PL4) and brown (PL2 and PL3) seeds, respectively. Of the DAMs in each pairwise comparison, over 92.5% were up-regulated in white seeds. Supportively, we found that white seeds showed a high relative content of organic acids, amino acids and derivatives, phenolic acids, saccharides and alcohols, nucleotides and derivatives, and lipids compared to brown seeds. Meanwhile, only the relative content of flavonoids was higher in brown seeds than in white seeds. These results indicate that the metabolite profile of perilla seeds is diverse according to varieties and seed coat colors. The correlation between seed coat color and variation in seed metabolite profile has been reported in many crops, including soybean, rice, sesame, and sorghum [16,18,32,34]. Moreover, the higher flavonoid content of brown perilla seeds is consistent with previous studies on sesame [16], soybean [35], rice [36], and bean [37], which found that the flavonoid content of dark seeds is higher than light seeds. Together, these findings denote that the biological properties of perilla seeds may vary depending on the seed coat color. The variation in the relative content of key bioactive DAMs suggests that white perilla seeds may possess more diversified and marked pharmacological abilities than brown seeds. Supportively, although both seeds showed higher antioxidant activities, seeds in the PL4 group activities were the lowest. Further pharmacological studies are required to thoroughly dissect the relationship between seed phenotypes and biological activities.

Beside seed coat color, we found that leaf color contributed significantly to variation in seeds’ metabolite profiles. Leaf color is a critical agronomic trait, and its variation significantly affects global plant metabolism [38,39]. We identified 68 (37 up-regulated in PL1) and 86 (35 up-regulated in PL3) DAMs in PL1. vs. PL4 and PL3. vs. PL2, respectively. The classification and functional annotation of DAMs showed that amino acids, organic acids, phenylpropanoids, saccharides, and nucleotides were the major differentially regulated metabolites in the different perilla seeds. These results infer that leaf color may affect the photosynthetic efficiency, resulting in different growth and yield performance and the observed diversity in metabolite profiles [38,39]. A number of DAMs were assigned to ABC transporters, confirming the diversity of primary metabolites transferred from leaves to the seeds in each type of perilla. An integrated dynamic analysis of the transcriptome and metabolome of developing leaves and seeds may provide deep insights into perilla plant physiology and metabolism, which in turn would contribute to developing novel perilla varieties for specific applications.

Chemical markers are essential for authenticating crop species, evaluating the quality of raw materials and finished products, and discriminating between plant organs from the same or different varieties [40]. Guan et al. selected rosmarinic acid, rosmarinic acid-3-*O*-glucoside, apigenin, and luteolin as perilla biomarkers [24]. In the present study, we identified sixteen key DAMs. These metabolites may represent potential biomarkers for discriminating among perilla seeds of different colors. Further quantification and analysis of these metabolites in diverse perilla genotypes are required to detect the ones to be used as discriminatory biomarkers specifically.

## 5. Conclusions

In summary, this study revealed that the perilla seed metabolite profile diverges considerably according to the seed coat color and leaf color. Light perilla seeds contain higher levels of diverse classes of metabolites, such as lipids, amino acids and derivatives, nucleotides and derivatives, phenolic acids, organic acids, saccharides and alcohols, and terpenoids, compared to brown seeds. In contrast, the brown seeds had the highest relative content of flavonoids. Key DAMs and associated pathways were identified, providing valuable resources for future studies to better understand perilla plant physiology and metabolism. Most key bioactive DAMs, including flavonoids (sophoricoside, quercetin-3’-*O*-glucoside, prunin, naringenin, naringenin chalcone, butin, genistin, kaempferol-3-*O*-rutinoside, hyperoside, etc.), amino acids (alanine, valine, serine, proline, lysine, histidine, glutamine, threonine, ornithine, and glutaric acid), and vitamins (B1, B3, B6, U, etc.), exhibited the highest relative content in PL3 (brown seed, purple leaf), PL1 (white seed, green-purple leaf), and PL4 (white seed, green leaf), respectively. Meanwhile, key differentially accumulated phenolic acids (rosmarinic acid-3’-*O*-glucoside, isovanillic acid, 2-hydroxycinnamate, 4-hydroxy-3-methoxycinnamic acid, 5-*O*-caffeoylshikimic acid, caffeic acid *O*-glucoside, isoacteoside, *p*-coumaric acid, vanillic acid, ferulic acid, etc.) showed a higher relative content in PL1 and PL4 than other groups. Furthermore, we identified sixteen potential biomarkers for discriminating between perilla seeds of different colors and found that both seed had higher antioxidant activities. But the antioxidant activities of the PL4 group members were the lowest. Our findings may represent fundamental bases for the comprehensive use of perilla seeds of different phenotypes.

## Figures and Tables

**Table 1:** List and phenotype characteristics of the twelve perilla varieties used in this study.

Variety Name	Sample ID	Sample Group	Seed Coat Color	Leaf Color
Zisu1	PL01	PL1	White	Green/purple
Zisu2	PL02		White	
Zisu3	PL04		White	
Baisu1	PL07	PL2	Brown	Green
Baisu2	PL08		Brown	
Baisu3	PL09		Brown	
Zisu4	PL06	PL3	Brown	Purple
Zisu5	PL10		Brown	
Zisu6	PL11		Brown	
Baisu4	PL03	PL4	White	Green
Baisu5	PL05		White	
Baisu6	PL12		White	

## Data Availability

Data is contained within the article and Supplementary Materials.

## Supplementary Materials

The following supporting information can be downloaded at: https://www.mdpi.com/article/10.3390/foods12234370/s1, Figures S1 and S2: Volcano plots of DAMs in pairwise comparisons; Figure S3: KEGG annotations and enrichment results of the DAMs in four pairwise comparisons; Figure S4: KEGG annotations and enrichment results of the DAMs in other pairwise comparisons; Figure S5: Relative content of key differentially accumulated bioactive alkaloids, quinones, and terpenoids; Table S1: List of the 975 identified metabolites in perilla seeds with their average relative content in each variety; Table S2: List of the sixteen key DAMs between perilla seeds of different coat colors; Table S3: List of DAMs between PL1 and PL4; Table S4: List of DAMs between PL3 and PL2.
