# Supplementary material for: Comparative Metabolomics Analysis of Different Perilla Varieties Provides Insights into Variation in Seed Metabolite Profiles and Antioxidant Activities
Source: Foods. 2023 Dec 4;12(23):4370. doi: 10.3390/foods12234370 (PMC10706770; doi:10.3390/foods12234370)
Supplement: Supplementary file 1 [file foods-12-04370-s001.zip › Supplementary Figures.pdf]

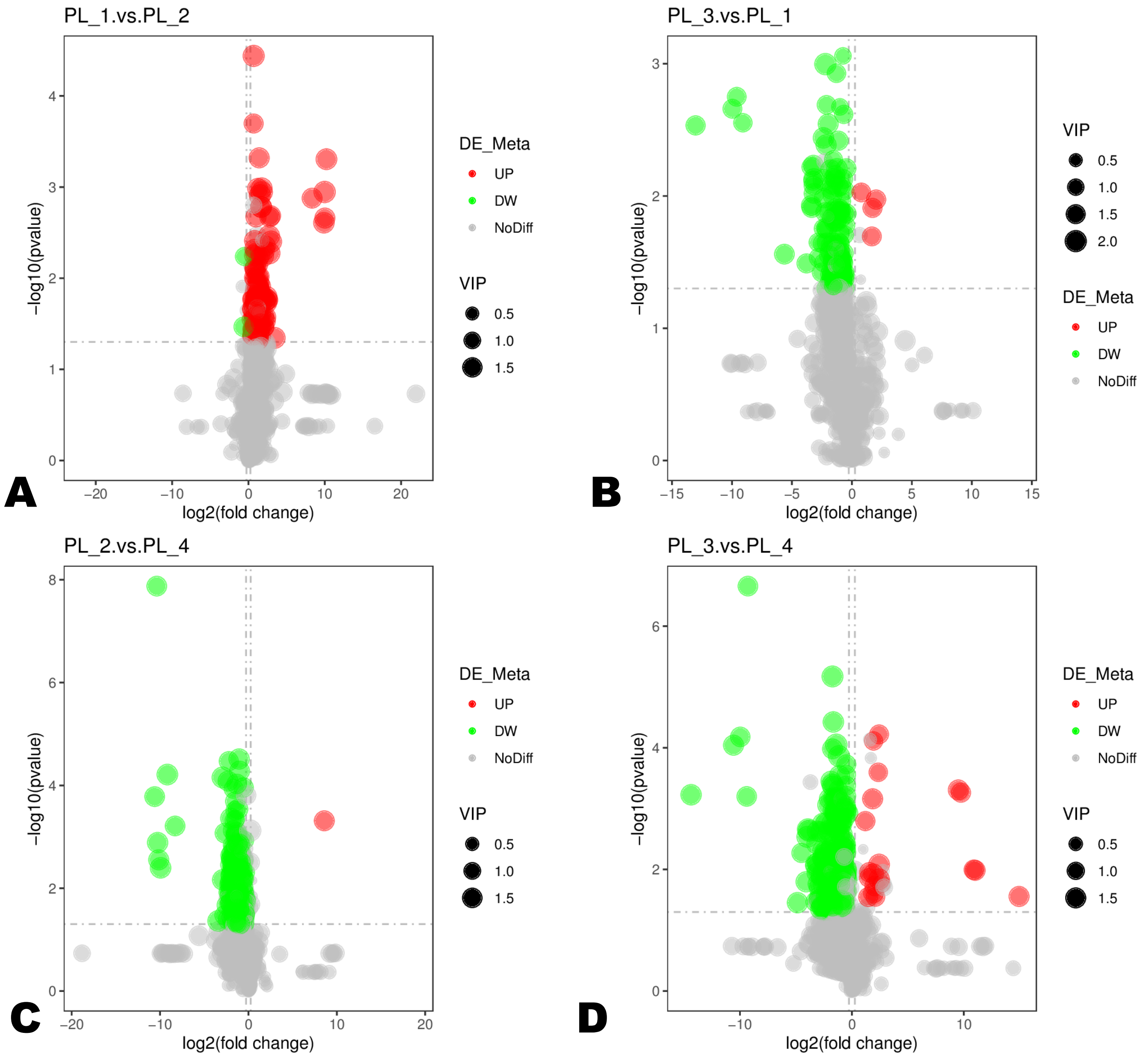

Figure S1. Volcano plots of DAMs in pairwise comparisons between: (A) PL1 and PL2; (B) PL3 and PL1; (C) PL2 and PL4; and (D) PL3 and PL4. The sample labels are defined in Table 1.

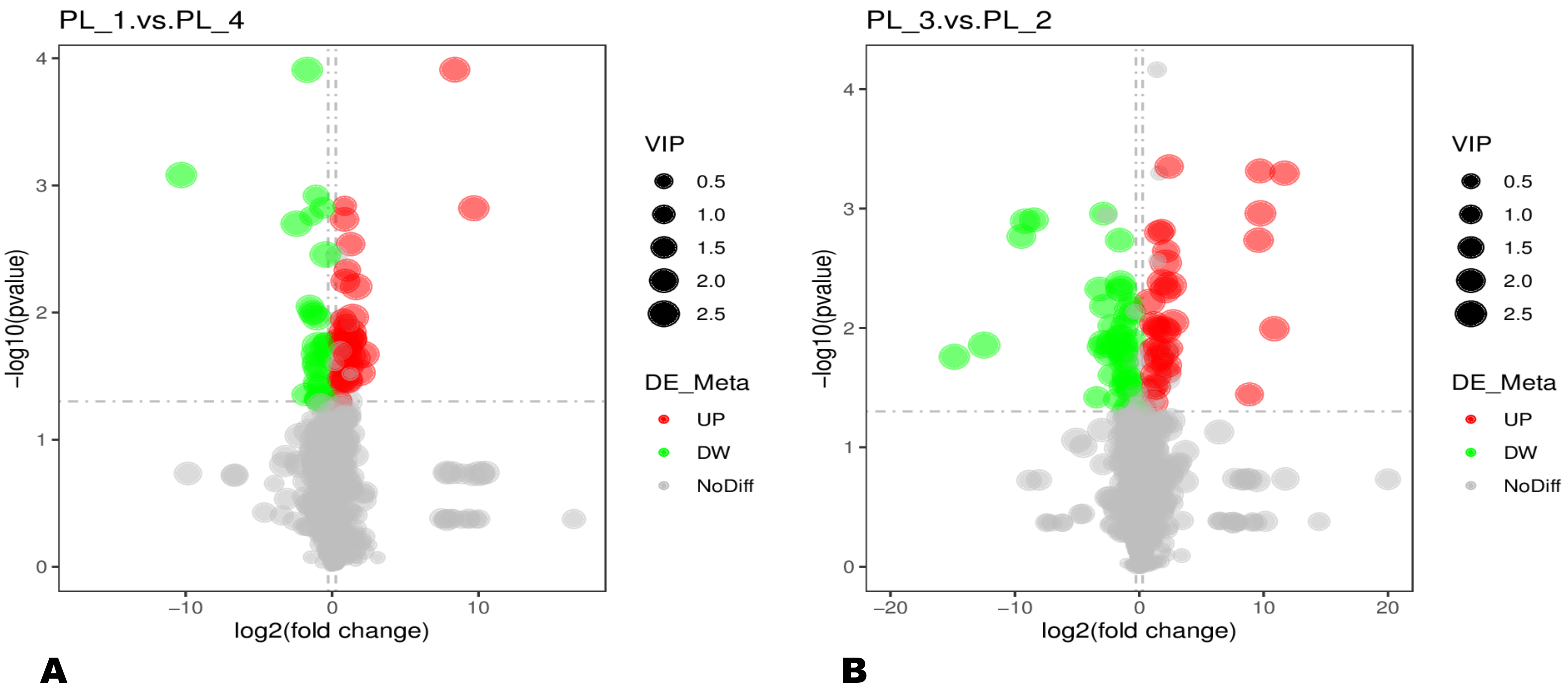

Figure S2. Volcano plots of DAMs in pairwise comparison between PL1.vs.PL4 (A) and PL3.vs.PL2 (B). The sample labels are defined in Table 1.

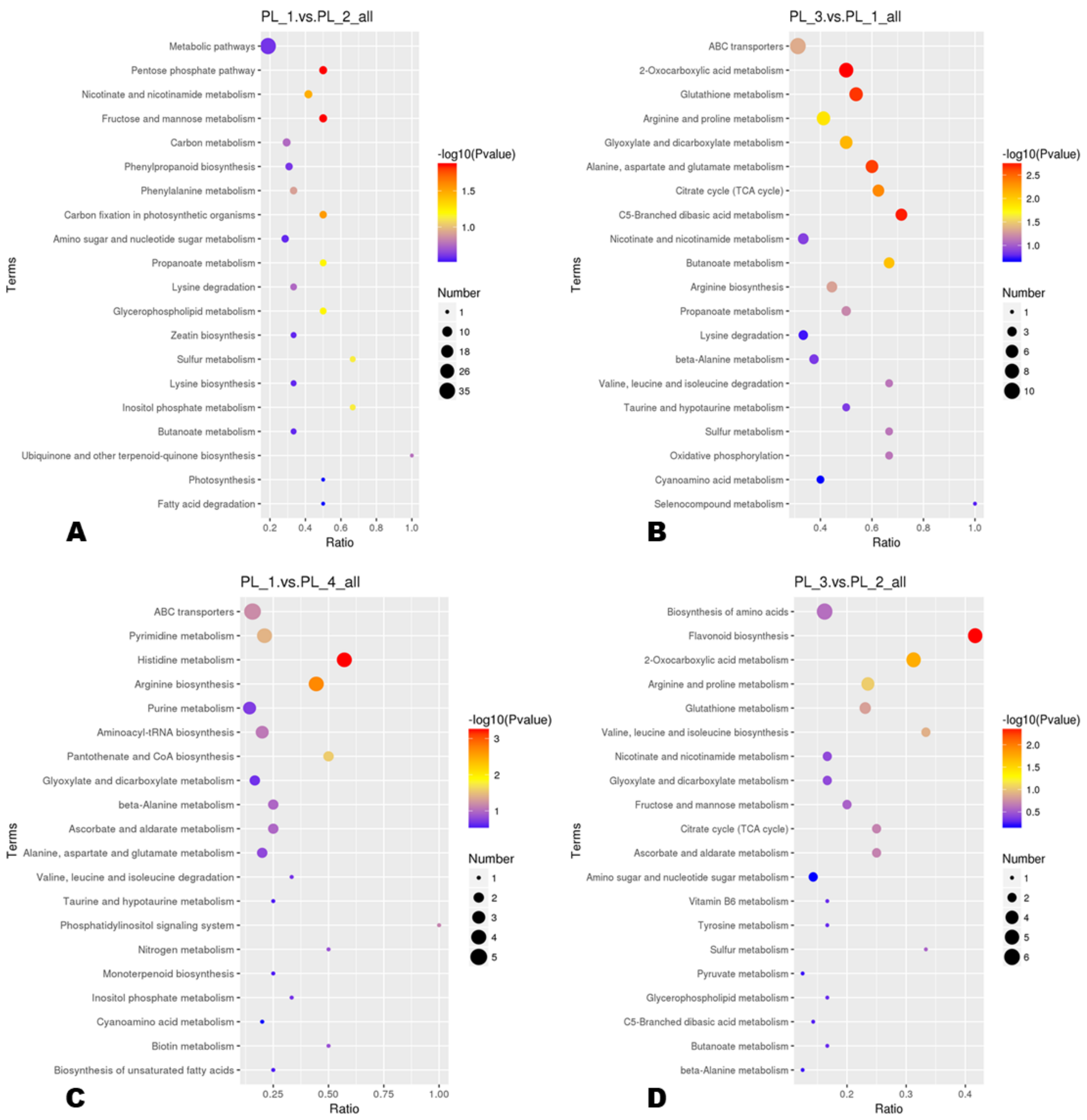

Figure S3. KEGG annotations and enrichment results of the DAMs in pairwise comparison between: (A) PL1.vs.PL2, (B) PL3.vs.PL1, (C) PL1.vs.PL4, and (D) PL3.vs.PL2 (B). The sample labels are defined in Table 1.

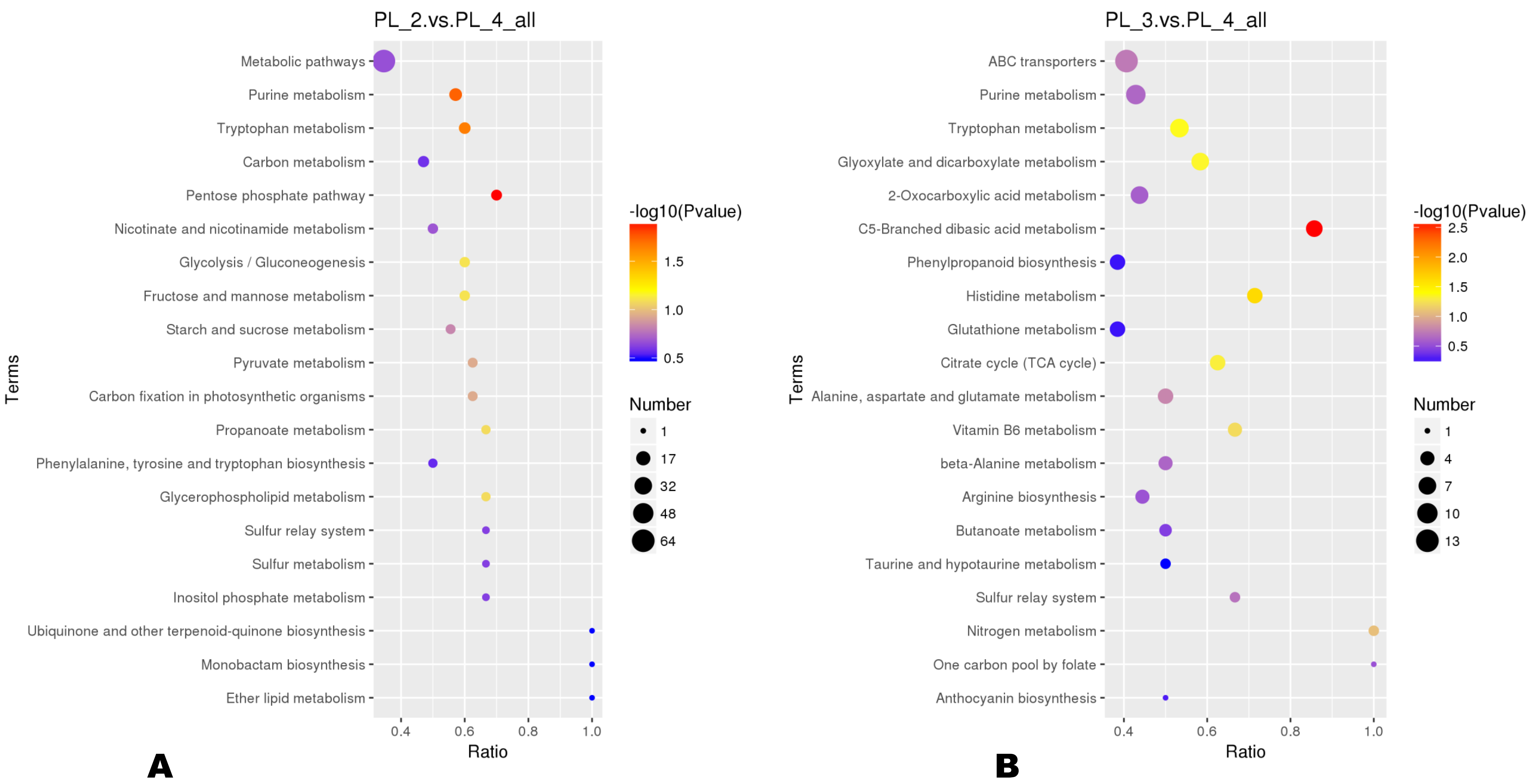

Figure S4. KEGG annotations and enrichment results of the DAMs in pairwise comparison between PL2.vs.PL4 (A) and PL3.vs.PL4 (B). The sample labels are defined in Table 1.

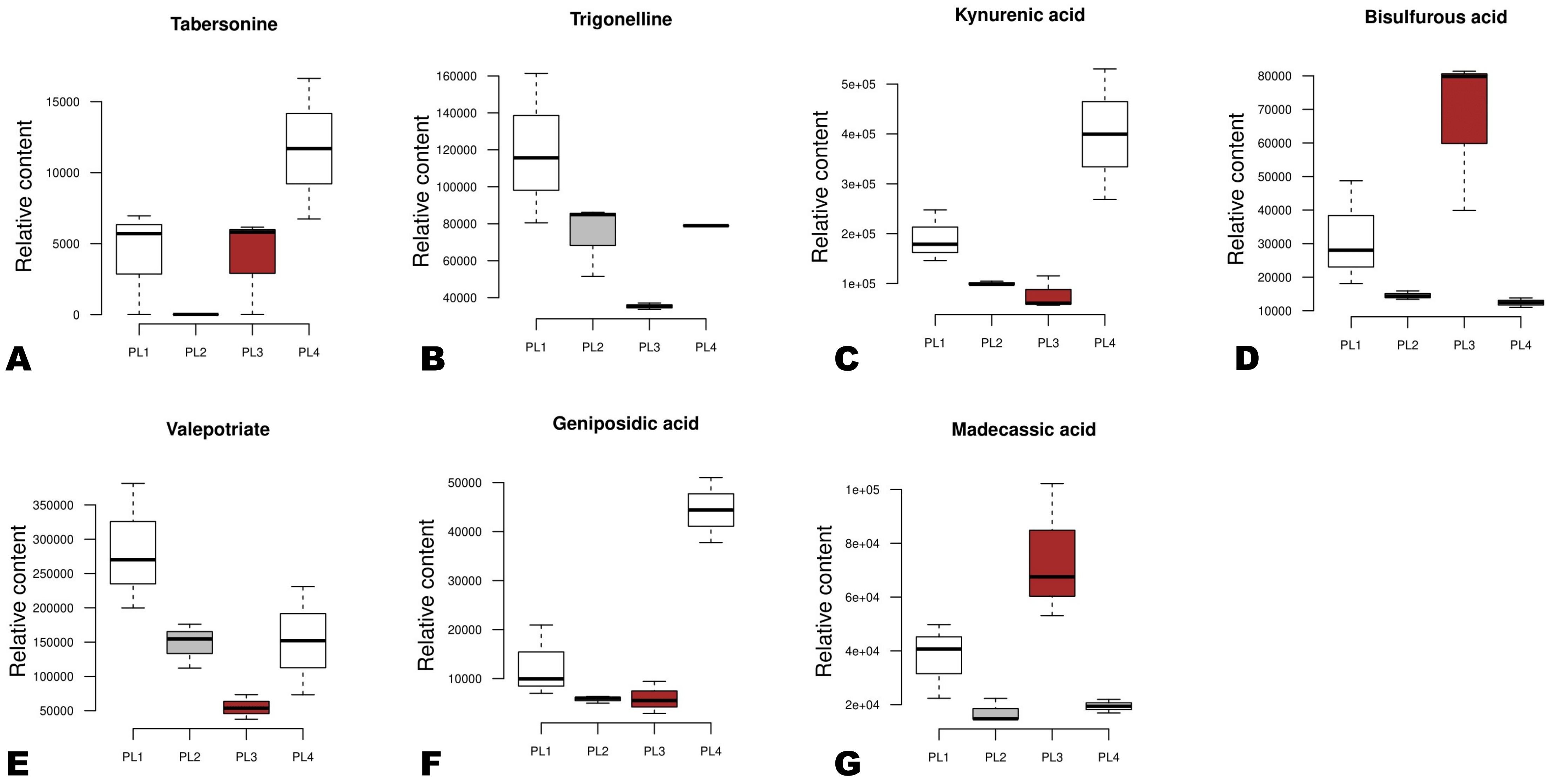

Figure S5. Relative content of key differentially accumulated bioactive alkaloids (A and B), quinones (C and D), and terpenoids (E-G) in the different perilla seeds. The sample labels are defined in Table 1.
